# Chitin Biodegradation by Lytic Polysaccharide Monooxygenases from *Streptomyces coelicolor* In Vitro and In Vivo

**DOI:** 10.3390/ijms24010275

**Published:** 2022-12-23

**Authors:** Fei Li, Honglu Zhao, Yuxin Liu, Jiaqi Zhang, Hongbo Yu

**Affiliations:** 1 Department of Bioengineering, School of Chemistry and Chemical Engineering, Wuhan University of Science and Technology, Wuhan 430081, China; 2 Department of Biotechnology, College of Life Science and Technology, Huazhong University of Science and Technology, Wuhan 430074, China

**Keywords:** lytic polysaccharide monooxygenases, transcription, expression, *Streptomyces coelicolor* A3(2), chitin biodegradation

## Abstract

Lytic polysaccharide monooxygenases (LPMOs) have the potential to improve recalcitrant polysaccharide hydrolysis by the oxidizing cleavage of glycosidic bond. *Streptomyces* species are major chitin decomposers in soil ecological environments and encode multiple *lpmo* genes. In this study, we demonstrated that transcription of the *lpmo* gene, *Sclpmo*10G, in the *Streptomyces coelicolor* A3(2) (*Sc*A3(2)) strain is strongly induced by chitin. The *Sc*LPMO10G protein was further expressed in *Escherichia coli* and characterized in vitro. The *Sc*LPMO10G protein showed oxidation activity towards chitin. Chitinase synergy experiments demonstrated that the addition of *Sc*LPMO10G resulted in a substantial in vitro increase in the reducing sugar levels. Moreover, in vivo the LPMO-overexpressing strain *Sc*ΔLPMO10G(+) showed stronger chitin-degrading ability than the wild-type, leading to a 2.97-fold increase in reducing sugar level following chitin degradation. The total chitinase activity of *Sc*ΔLPMO10G(+) was 1.5-fold higher than that of *Sc*A3(2). In summary, *Sc*LPMO10G may play a role in chitin biodegradation in *S. coelicolor*, which could have potential applications in biorefineries.

## 1. Introduction

Chitin is one of the most abundant renewable polysaccharides and is found in the exoskeletons and cell walls of many organisms. Chitin consists of N-acetylglucosamine units interconnected by β-1,4 glycosidic bonds and exists as crystals [1]. The complex structure of chitin can effectively prevent abiotic and biological degradation; however, this also limits the development and utilisation of biomass. The biodegradation processes of recalcitrant polysaccharide materials, such as chitin, were initially thought to solely refer to polysaccharide hydrolases [2,3,4,5,6]. However, the discovery of lytic polysaccharide monooxygenases (LPMOs) has revolutionised this view. Vaaje-Kolstad et al. first described that chitin-binding protein 21 (CBP21) could oxidise crystalline chitin and enhance its depolymerization by chitinases [7]. LPMOs can lead to subsequent cleavage of the glycosidic bonds on the surface of the polysaccharide through the oxidation of various carbons (e.g., C1 and C4) by Cu-superoxide or Cu-oxyl intermediates, which generates oxidized and non-oxidized chain ends. The reaction involves a divalent copper ion within the active site, in addition to molecular oxygen/hydrogen peroxide and externally provided electrons to potentiate LPMOs activity [8,9]. The oxidative cleavage of glycosidic bonds in crystalline polysaccharides by LPMOs improves the accessibility of substrates for glycoside hydrolases, thereby enhancing the overall efficiency of recalcitrant polysaccharides using complex enzymatic systems [7,10,11,12].

LPMOs are now classified as auxiliary activities (AA) and grouped into eight families (AA9–11, AA13–17) in the CAZy database (http://www.cazy.org/Auxiliary-Activities.html/ (accessed on 4 March 2022)) [7,13,14,15,16,17,18]. LPMOs are widespread and highly abundant in the fungal and bacterial genomes, and more than 20 *lpmo* genes have been identified in the genomes of some ascomycetous and basidiomycetous fungi [19]. It is unclear whether all *lpmo* genes from the same AA family in fungi or bacteria genomes exhibit the same functions and act on similar substrates, and whether these genes are induced and expressed under the same substrate conditions.

*Streptomyces* species are the most widely distributed bacteria in soil and are important microbial contributors to biomass decomposition in the carbon cycle [20,21]. Actinomycetes have evolved a complex enzyme system to obtain soluble nutrients from chitin [20]. Seven putative LPMOs in the AA10 family are encoded by the *S. coelicolor* A3(2) (ScA3(2)) genome. These LPMOs have been heterologously expressed in *Escherichia coli* and identified as having in vitro chitin or cellulose-oxidizing activity [22,23,24,25,26]. Previous studies have shown the enzymatic properties and functional significance of LPMOs in *S. coelicolor* when acting on cellulose. The LPMO CelS2 has been described to be co-expressed with cellulase [27] and exhibits C1-oxidizing activity on crystalline cellulose, thereby acting in synergy with cellulases [24]. In addition, *Sc*LPMO10B displays C1/C4-oxidizing activity, which can compensate for CelS2 activity and synergistically degrade cellulose [22]. There are only few reports on the expression patterns of the *lpmo* genes during chitin depolymerisation by *Sc*A3(2). It was also found that *Sc*LPMO10B demonstrates oxidizing activity on chitin [22]; however, the key *lpmo* gene acting on chitin in *Sc*A3(2) is also unknown. Additionally, there remains a lack of in vivo studies on the biological function of LPMOs in chitin depolymerisation by *Sc*A3(2).

In this study, we hypothesize that the expression of LPMO in vivo is relative to the substrate, and overexpression of LPMO in the original host could enhance substrate degradation. We examined the relative transcript levels of *lpmo* during chitin depolymerization by *Sc*A3(2). The LPMO with the highest relative transcript level (*Sc*LPMO10G) was expressed, purified, and characterised in vitro. Further, LPMO-overexpressing mutant strain *Sc*ΔLPMO10G(+) was used to study the degradation of chitin in vivo. These results, for the first time, provide evidence for the function of LPMOs in *S. coelicolor* for the in vitro and in vivo biodegradation of chitin and further contribute to the understanding of the mechanisms and application of bacterial biodegradation of recalcitrant polysaccharides.

## 2. Results and Discussion

### 2.1. Transcript Level of lpmo Genes in the Presence of Chitin

**Figure 1:**
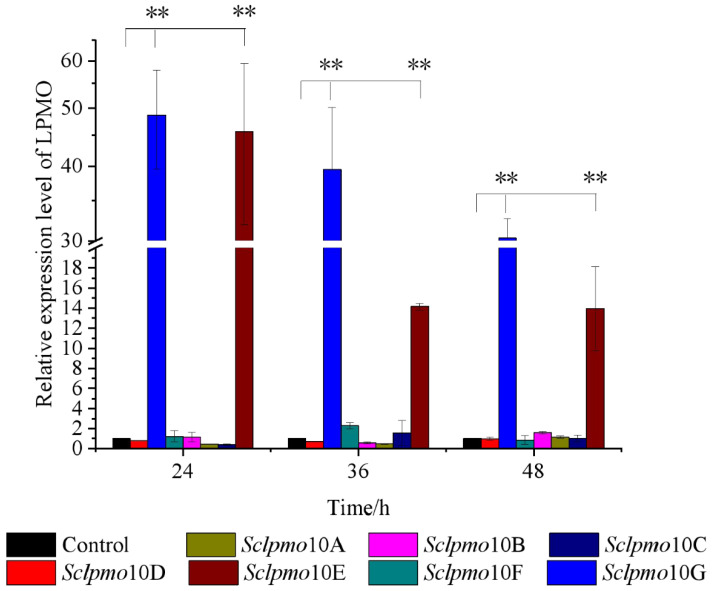
Time course of relative transcript level of *Sclpmo*s in the presence of 0.1% chitin. Relative expression level of *Sclpmo*s cultured with glucose as control. All experiments were performed in triplicate and standard deviation analysis was conducted (SD, *n* = 3). Asterisks indicate significant differences (*p* < 0.01).

To determine the chitin-induced responses of *lpmo* genes in *S. coelicolor*, RNA samples in the presence of chitin and glucose were extracted at various time points, and the transcription levels of 7 *lpmo* genes were examined using qPCR analysis, with *hrd*B internal reference [28,29]. Samples cultured with glucose as the control were used to calculate the relative transcriptional levels. The results indicated that, in the presence of 0.1% chitin, the relative transcription levels of *Sclpmo*10E and *Sclpmo*10G were significantly higher than those cultured with glucose (*p* < 0.01), whereas the other 5 *lpmo* genes did not show a significant response to chitin (Figure 1). The differential expression patterns between *Sclpmo*10G and other *Sclpmo*10s indicate that the expression of LPMO is related to the substrate. The expression of different LPMOs can be induced by substrates, and different LPMOs could have different functions.

*Sclpmo*10G exhibited the highest relative transcript level during the entire induction period, with chitin as the sole carbon source. The relative transcript levels of *Sclpmo*10E and *Sclpmo*10G gradually decreased with increasing induction time; however, the relative transcript level of *Sclpmo*10G remained at a higher level. The relative transcript levels of *Sclpmo*10G in chitin cultures increased by 48.7-, 39.5-, and 30.3-fold after incubation for 24, 36, and 48 h, respectively, compared to those in glucose cultures. Although the relative transcript level of *Sclpmo*10E increased by 45.6-fold after 24 h, it decreased significantly to approximately 31% of that after 24 h following incubation for 36 h. The transcript level of a gene is usually affected by different growth stage of cells and the external environment. Through gene expression regulation, *S. coelicolor* can express a large number of *Sc*LPMO10E and *Sc*LPMO10G proteins to adapt to the growing environment in the presence of chitin. A similar phenomenon has been reported by Nazari et al. [28], who found that in the presence of colloidal chitin, chitinase genes in soil were transcriptional inducted at different levels and the highest levels of induction were obtained after 48 h incubation. These results suggest that *Sclpmo*10G plays an important role in chitin degradation by *S. coelicolor*. Therefore, we further investigated the *Sc*LPMO10G protein to explore the function of *Sclpmo*10G in chitin degradation.

### 2.2. Expression and Activity Assay of ScLPMO10G

To achieve expression of *Sclpmo*10G, the structural gene of *Sclpmo10G* was cloned into the expression vector pET28a and transferred into *Escherichia coli* Rosetta(DE3). SDS-PAGE analysis showed the purified protein *Sc*LPMO10G was obtained as a band of approximately 25 kDa using a Ni-NTA column, which is consistent with the predicted molecular weight of *Sc*LPMO10G (Supplementary Figure S1).

**Figure 2:**
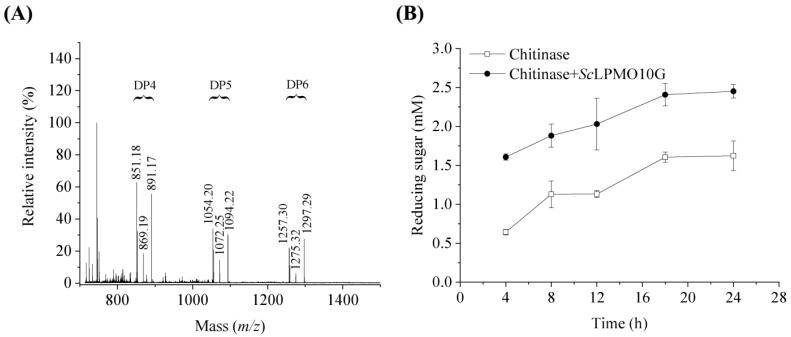
Activity of *Sc*LPMO10G on chitin. (**A**) Chitin degradation products by *Sc*LPMO10G activity identified using MALDI TOF MS analysis. DP indicates the degree of polymerisation of oxidized oligosaccharides. Possible products in these clusters are the sodium adducts of the lactone (*m*/*z* 851.18, 1054.20, 1257.30), the sodium adducts of the aldonic acid (*m*/*z* 869.19, 1072.25, 1275.32), and the sodium adduct of the aldonic acid sodium salt (*m*/*z* 891.17, 1094.22, 1297.29). 100% relative intensity represents 1.9 × 10^4^ arbitrary units (a.u.) for full spectra. (**B**) Degradation of 10.0 mg/mL chitin by 0.2 μM chitinases in the presence (solid circle) or absence (hollow square) of 2 μM *Sc*LPMO10G, with ascorbic acid (1.0 mM) used as the electron donor. All experiments were performed in triplicate and standard deviation analysis was conducted (SD, *n* = 3).

To identify the activity of *Sc*LPMO10G *on chitin*, the reaction products were detected using MALDI-TOF MS (Figure 2A). The glycosidic bonds in chitin were broken and generated oxidized chain ends through C1 oxidation. Soluble products of aldonic acid metabolism with different degrees (4, 5, and 6) of polymerisation were generated from chitin, indicating that *Sc*LPMO10G was active against chitin. Possible products in these clusters are the sodium adducts of the lactone (*m*/*z* 851.18, 1054.20, 1257.30), the sodium adducts of the aldonic acid (*m*/*z* 869.19, 1072.25, 1275.32), and the sodium adduct of the aldonic acid sodium salt (*m*/*z* 891.17, 1094.22, 1297.29). No oligomerization products of chitin were observed when the electron-donating ascorbic acid was not added. Further, the addition of *Sc*LPMO10G promoted chitin degradation by chitinase and increased the production of reducing sugars. The addition of *Sc*LPMO10G (2 μM) resulted in the production of 1.5 times more reducing sugars than in the condition without *Sc*LPMO10G (Figure 2B). This might be caused by the chain break introduced by *Sc*LPMO10G, which increases the substrate accessibility for chitinases [7,25,30]. *Sc*LPMO10G exerts no oxidative activity against cellulose substrate according to the MALDI-TOF MS analysis (Supplementary Figure S2).

### 2.3. Effect of pH and Temperature on ScLPMO10G Activity and Stability

**Figure 3:**
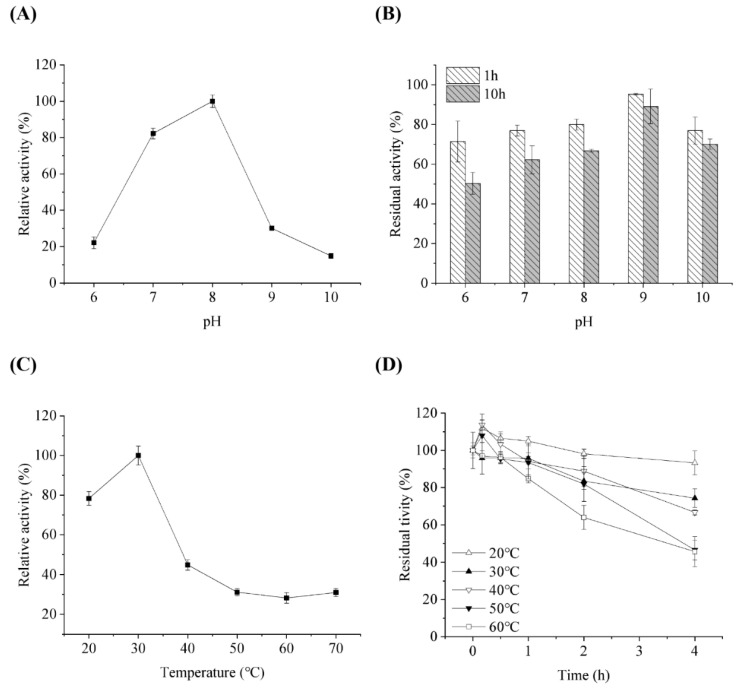
Influence of pH on enzymatic activity (**A**) and stability (**B**) of *Sc*LPMO10G against 2,6-DMP. For (**A**,**B**), the activity at pH 8 and initial activity were set at 100%, respectively. Influence of temperature on enzymatic activity (**C**) and stability (**D**) of *Sc*LPMO10G against 2,6-DMP. For (**C**,**D**), the activity at 30 °C and initial activity were set at 100%, respectively. All experiments were performed in triplicate and standard deviation was analysed (SD, *n* = 3).

The effects of pH and temperature on *Sc*LPMO10G activity and stability were determined. The data showed that the optimum pH for the enzyme to oxidize 2,6-DMP was 8.0 (Figure 3A), and that it was more stable under alkaline conditions. Approximately 90% residual activity was retained after incubation at pH 9.0 for 8 h (Figure 3B). The optimal pH of *Sc*LPMO10G was similar to that of *Ba*tLPMO10 from *Bacillus subtilis* and some AA9 LPMO; however, it was more stable than AA9 proteins with less than 60% residual activity under alkaline conditions (pH 9.0) [31,32]. The optimum temperature for *Sc*LPMO10G activity was found to be 30 °C (Figure 3C). *Sc*LPMO10G was more stable at a temperature range of 20 to 40 °C. After treatment at 20, 30, and 40 °C for 4 h, the residual activity of *Sc*LPMO10G was 93.3, 74.3, and 66.7%, respectively; however, over 50% activity was lost following incubation over 60 °C (Figure 3D). Compared to that of *Sc*LPMO10C from *S. coelicolor* of which the activity disappeared completely after 20 min of incubation at 40–60 °C, *Sc*LPMO10G showed better thermostability [33].

### 2.4. Chitin Degradation by the LPMO Overexpression Mutant Strain ScΔLPMO10G(+)

**Figure 4:**
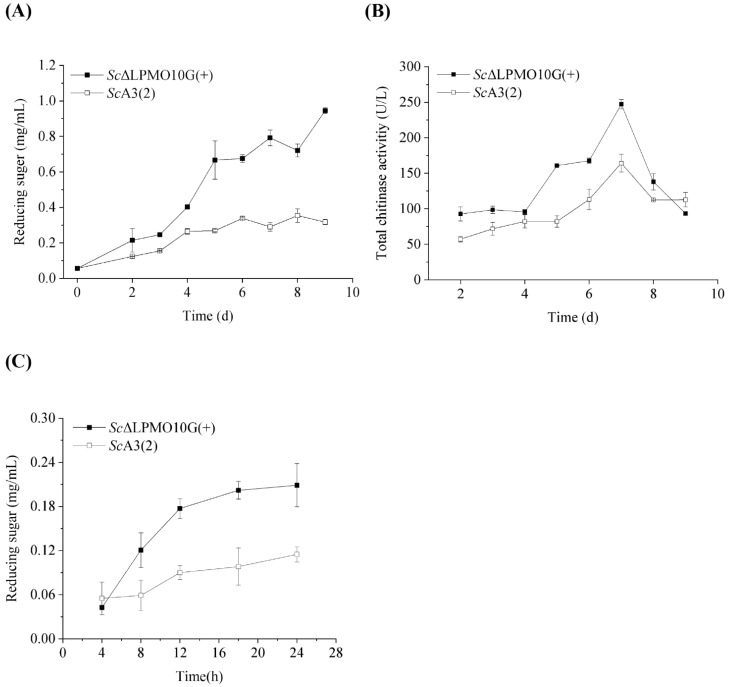
Chitin degradation by wild-type and the *Sc*ΔLPMO10G(+) mutant strains. (**A**) The reducing sugar concentration of the culture supernatant during the incubation of wild-type *Streptomyces coelicolor* A3(2) (*Sc*A3(2)) and its mutant strain *Sc*ΔLPMO10G(+). The strains were cultivated on MM medium using chitin as the sole carbon source at 28 °C. (**B**) The total chitinase activity of *Sc*A3(2) and its mutant strain *Sc*ΔLPMO10G(+). (**C**) Chitin degradation by extracellular enzyme produced by the wild type *Sc*A3(2) and its mutant strain *Sc*ΔLPMO10G(+). All experiments were performed in triplicate and standard deviation was analysed (SD, *n* = 3).

The transcription levels of *Sclpmo*10G were significantly increased by 461.9-fold in the mutant strain *Sc*ΔLPMO10G(+) than the wild-type strain, which confirmed that *Sc*LPMO10G was over-produced in the mutant strain compared to that in the wild-type (Supplementary Figure S3A). The *Sc*LPMO10G protein was also significantly higher in ScΔLPMO10G(+) than ScA3(2), highlighting the overexpression of *Sc*LPMO10G in mutant strain *Sc*ΔLPMO10G(+) (Supplementary Figure S3B). Chitin degradation by *Sc*A3(2) and *Sc*ΔLPMO10G(+) was analysed to identify the function of *Sc*LPMO10G. The reducing sugar concentration and enzyme activity of intercellular supernatant were examined to estimate the ability of the wild-type and mutant strains to degrade chitin. The reducing sugar concentration gradually increased with incubation time, and the mutant strain *Sc*ΔLPMO10G(+) yielded more reducing sugars than the wild-type strain *Sc*A3(2) when chitin was used as the carbon source (Figure 4A). The accumulation level of reducing sugar reached 0.95 mg/mL in the mutant strain *Sc*ΔLPMO10G(+), which was approximately 2.97-fold higher than that in the wild-type, following nine days of incubation. The increased mRNA transcription (461.9-fold) seemingly led to a small reward in the releasing sugar (2.97-fold higher), which could be related to weak protein secretion ability and an inappropriate ratio of LPMOs to chitinase expression.

The activity of chitinase secreted by *Sc*A3(2) and *Sc*ΔLPMO10G(+) displayed a similar trend over time, as previously reported [34]. However, the chitinase activity of *Sc*ΔLPMO10G(+) was significantly higher than that of the wild-type strain during the entire cultivation period (Figure 4B). After seven days of incubation, the highest chitinase activity level for *Sc*ΔLPMO10G(+) and *Sc*A3(2) reached 247.2 and 164.3 U/L, respectively, resulting in a higher proportion of reducing sugars being released by *Sc*ΔLPMO10G(+), as described above. The releasing sugar content is gradually increasing with the increase of culture time (Figure 3A). This was consistent with the activity of chitinase secreted by ScA3(2) and ScΔLPMO10G(+) (Figure 3B). Furthermore, 0.1 mg/mL of extracellular enzymes with the highest chitinase activity were used to degrade chitin under in vitro conditions (Figure 4C). Compared to *Sc*A3(2), the extracellular enzymes from *Sc*ΔLPMO10G(+) showed a stronger ability to depolymerise crystalline chitin. Further, the reducing sugar content in *Sc*ΔLPMO10G(+) was approximately 1.8-fold greater than that of *Sc*A3(2), after 24 h of incubation. These results indicate that the in vivo overexpression of *Sc*LPMO10G enhances chitin degradation by *Sc*A3(2), and that *Sc*LPMO10G plays a significant role in the chitin biodegrading behaviour of *Sc*A3(2).

## 3. Materials and Methods

### 3.1. Plasmids, Strains, and Medium

Chitin (C_8_H_13_NO_5_)_n_ from shrimp shells was purchased from Aladdin (Shanghai, China). *Sc*A3(2) was activated for spore germination on a fresh mannitol soya flour (SFM) medium (20 g/L soybean powder was boiled for 3 h and filtered, the filtrate was mixed with 20 g/L mannitol and 20 g/L agar, and then autoclaved at 121 °C for 30 min) at 28 °C for 5–7 days, and the spores were collected and cultured in a tryptone soya broth (TSB) medium (17 g/L tryptone, 3 g/L tryptone soya broth, 5 g/L NaCl, 2.5 g/L K_2_HPO_4_, and 2.5 g/L glucose) for culturing *S. coelicolor* strains. A minimal medium (MM) (2.62 g/L K_2_HPO_4_·3H_2_O, 2 g/L KH_2_PO_4_, 2 g/L KNO_3_, 0.024 g/L MgSO_4_, 0.1 g/L yeast extract, and 1 g/L glucose) was used to explore the transcript levels of the *lpmo* genes from *S. coelicolor* in response to chitin. The LPMO-overexpressing mutant strain *Sc*ΔLPMO10G(+) was cultured and induced in a yeast extract-malt extract (YEME) medium (5 g/L tryptone, 3 g/L yeast extract, 3 g/L malt extract, 10 g/L glucose, and 340 g/L sucrose) to produce the *Sc*LPMO10G protein, with 25 μg/mL thiostrepton as an inducer. *Escherichia coli*, ET12567/pUZ8002, was used to introduce vectors into *Sc*A3(2) via conjugation. The vector pGM1190, carrying the *tip*A promoter, was used for homologous overexpression in *S. coelicolor*. Tryptone, tryptone soya broth, yeast extract and malt extract were purchased from OXOID (UK) and the other reagents were purchased from Sinopharm (Beijing, China).

### 3.2. The Relative Transcript Level of lpmo from S. coelicolor in Chitin Degradation

The *S. coelicolor* was cultured in the YEME medium at 28 °C and 200 rpm for 2–4 days. The cultures of *S. coelicolor* were collected, washed three times, and inoculated in the MM medium containing 1 g/L chitin or glucose until OD_600_ reached approximately 1.0. Samples were collected at 24–48 h post inoculation and subjected to quantitative polymerase chain reaction (qPCR). Total RNA was extracted from *S. coelicolor* using a bacterial RNA kit (Omega Bio-Tek, Beijing, China) and reverse transcribed using the HiScript II Q Select RT SuperMix for qPCR (+gDNA wiper) Kit (Vazyme, Nanjing, China). The relative transcript levels of the *lpmo* genes were examined using two-step real-time (RT)-PCR with ChamQ^TM^ SYBR qPCR Master Mix (Vazyme, Nanjing, China). The primers used for qPCR are listed in Table 1. The qPCR conditions were as follows: one cycle at 95 °C for 30 s, followed by 40 cycles of 10 s at 95 °C, 60 s at 60 °C, and a melting curve stage. All reactions were performed in triplicate. *hrdB* was selected as the internal reference gene to calculate the transcription levels of the *lpmo* genes [28,29].

### 3.3. Expression and Purification of ScLPMO10G

To express the *Sc*LPMO10G protein, signal peptides of the *Sc*LPMO10G (KEGG: SCO7225) were predicted by SignalP-5.0 (https://services.healthtech.dtu.dk/service.php?SignalP-5.0 (accessed on 5 March 2022)), indicating a 30-amino-acid signal peptide sequence for *Sc*LPMO10G. The *Sclpmo*10G gene encoding mature peptide was amplified by PCR using the primers *Sc*lpmo10G-EF and *Sclpmo*10G-R with a factor Xa cleavage site introduced and homologous sequence of the expression vector pET28a (Table 1) and ligated into NdeI/EcoRI-digested pET28a by the Gibson Assembly reaction with a histidine tag at the N-terminus of the protein, using the pEASY^®^-Basic Seamless Cloning and Assembly Kit (TransGen Biotech, Beijing, China).

The recombinant plasmid was cloned into *Escherichia coli* Rosetta(DE3) for expression. The combinant strain was cultivated in LB containing 30 μg/mL kanamycin at 37 °C and induced with 1 mM IPTG at 16 °C. The strains were centrifuged at 10,000× *g* for 5 min at 4 °C, resuspended in lysis buffer (20 mM imidazole and 0.5 M NaCl in 20 mM, pH 8.0 NaH_2_PO_4_-NaOH buffer), and then the samples were ultrasonicated. The supernatants were collected by centrifugation at 12,000× *g* for 30 min at 4 °C. The Ni-NTA column (Ni Sepharose 6 Fast Flow, GE Healthcare, Chicago, IL, USA) was used to purify the *Sc*LPMO10G protein, which was eluted with a buffer containing 0.5 M NaCl, 100 mM imidazole, and 20 mM NaH_2_PO_4_-NaOH (pH 8.0). Protein elution was collected and dialysed in 20 mM Tris-HCl buffer (pH 8.0), and then treated using a Factor Xa Cleavage Capture Kit (Novagen, Madison, WI, USA) to remove His-tag at 25 °C for 16 h. The purified *Sc*LPMO10G protein was saturated with CuSO_4_, following a previous report. The details are as follows: the purified *Sc*LPMO10G protein was saturated with a 3-fold molar excess of CuSO_4_ at room temperature, and then subjected to dialysis with 20 mM pH 7.0 Tris-HCl buffer at least three times. The protein solution obtained from dialysis was concentrated and further loaded onto a PD MidiTrap G-25 column to desalt according to the method reported by Loose et al. [35,36]. *Sc*LPMO10G purity was identified by sodium dodecyl sulphate-polyacrylamide gel electrophoresis (SDS-PAGE) analysis, and the protein concentration was measured using the Bradford micro-assay kit (Beyotime, Shanghai, China).

### 3.4. Activity Assay of ScLPMO10G

Chitin and phosphoric acid swollen cellulose (PASC) were used as the substrates to determine the activity of *Sc*LPMO10G. The reaction mixture comprised 2 μM *Sc*LPMO10G, 1 mM ascorbic acid, and 10 mg/mL substrate in 50 mM Tris-HCl buffer (pH 8.0). The reactions were incubated at 30 °C for 24 h. The chito-oligosaccharide aldonic acids generated by the action of *Sc*LPMO10G were identified using matrix-assisted laser desorption/ionization time-of-flight mass spectrometry (MALDI-TOF MS), as previously described [7,36].

### 3.5. Chitin Degradation by ScLPMO10G and Commercial Chitinase

The reaction mixtures contained 15 μg/mL chitinase (from *S. griseus*, Sigma-Aldrich, St. Louis, MO, USA), 10 mg/mL chitin as the substrate in the presence or absence of 2.0 μM *Sc*LPMO10G, with 1.0 mM ascorbic acid, and incubated in 50 mM Tris-HCl buffer (pH 8.0) at 37 °C according to our previously published study [37]. The samples were collected to measure the release of reducing sugars. The reducing sugars released from chitin by enzyme hydrolysis were determined using a 4-Hydroxybenzhydrazide (PAHBAH) colorimetry assay [38] at 410 nm. The concentration of reducing sugars was quantified by a standard curve with known concentrations of N-acetylglucosamine. All reactions were performed in triplicates.

### 3.6. Influence of Temperature and pH on Enzymatic Activity and Stability

LPMO activity was detected based on 2,6-dimethoxyphenol (2,6-DMP) oxidation, according to a previous report [39]. LPMO oxidises 2,6-DMP to form coerulignone with a high molar absorption coefficient (*ε*_469_ = 53,200 M^−1^ cm^−1^). To examine the influence of temperature on the activity and stability of *Sc*LPMO10G, reactions using 1 mM 2,6-DMP as the substrate, 2 μM *Sc*LPMO10G, and 0.1 mM H_2_O_2_ in 50 mM Tris-HCl buffer (pH 8.0) were performed at varying temperatures (20–70 °C). To evaluate the influence of pH on the activity and stability of *Sc*LPMO10G, reactions using 1 mM 2,6-DMP, 2 μM *Sc*LPMO10G, and 0.1 mM H_2_O_2_ incubated in pH 6–10 Britton–Robinson buffers at 30 °C were performed. The concentrations of coerulignone were measured at 469 nm.

### 3.7. Construction of LPMO Overexpression Mutant Strain

The gene encoding the *Sc*LPMO10G with a native peptide was amplified through PCR using the primers *Sclpmo*10G-SF and *Sclpmo*10G-R with a homologous sequence of the expression vector pGM1190 (Table 1). To construct the expression plasmid pGM1190-*Sclpmo*10G, the amplified fragment *Sc*LPMO10G was ligated into *Nde*I/*EcoR*I-digested pGM1190 by the Gibson Assembly reaction, using the *pEASY*^®^-Basic Seamless Cloning and Assembly Kit (TransGen Biotech, Beijing, China). The map and sequence of recombinant plasmid could be seen in Figure S1 and Table S1. The ligated product was transformed into *E. coli* DH5α cells, and the transformants were screened based on apramycin sulphate resistance and verified by sequencing. A thiostrepton-induced *tip*A promoter was used to control the expression of the downstream genes in this system. The desired pGM1190-*Sclpmo*10G plasmid was cloned into *E. coli* ET12567/pUZ8002 and subjected to intergeneric conjugation. Intergeneric conjugation of *E. coli* ET12567/pUZ8002 to *Sc*A3(2) was executed as previously described [40]. The conjugates were screened on SFM containing 25 μg/mL apramycin sulphate and nalidixic acid sodium, resulting in the formation of the LPMO-overexpressing mutant strain *Sc*ΔLPMO10G(+). To investigate the expression level of *Sc*LPMO10G in the overexpression mutant strain, the strain was induced with 25 μg/mL thiostrepton at 28 °C for 2–3 days and collected by centrifugation, and RNA extraction was performed and further subjected to qPCR analysis. The strain with an empty plasmid pGM1190 was set as a negative control.

### 3.8. Chitin Degradation by the LPMO Overexpression Mutant Strain ScΔLPMO10G(+)

To investigate the effect of *Sc*LPMO10G on in vivo chitin degradation, chitin degradation in the LPMO-overexpressing *Sc*ΔLPMO10G(+) strain was examined. SFM agar was used to collect the spores, which were inoculated in TSB medium with 25 mg/mL apramycin sulphate and grown for 2–3 days. The mycelia were then transferred to fresh TSB medium with an inoculation of 2% (*v*/*v*) for 2–3 days. Mycelia were harvested by centrifugation and resuspended in fresh TSB medium. Approximately 1% of the mycelia (*w*/*v*) were inoculated into MM containing 10 g/L chitin with 25 μg/mL apramycin sulphate. After cultivation for 2–3 days at 28 °C, the culture medium was added with 0.2 mM CuSO_4_ and 25 μg/mL thiostrepton to induce *Sc*LPMO10G expression. Total chitinase activity was determined by measuring the concentration of reducing sugars, according to a previous report [34]. One millilitre of reaction mixture containing 700 μL extracellular supernatant of the wild-type or its mutant strain *Sc*ΔLPMO10G(+), 300 μL 2% chitin was incubated for 2 h at 37 °C. The amount of enzyme required to catalyse the production of 1 mg N-acetylglucosamine per hour at 37 °C was defined as one activity unit (U) of chitinase. In addition, the extracellular enzyme from the mutant strain *Sc*ΔLPMO10G(+) was also further subjected to chitin degradation. The reaction mixture containing extracellular enzyme with 0.1 mg/mL proteins and 10 mg/mL chitin was incubated at 37 °C in 50 mM Tris-HCl buffer (pH 8.0). An inactivated extracellular protein prepared with a high temperature treatment at 121 °C for 20 min was used as the blank control. The concentration of reducing sugar released was determined based on the PAHBAH colorimetry assay, as described above.

## 4. Conclusions

In this study, we studied the transcription levels of *lpmo* genes in *Sc*A3(2) during chitin biodegradation and showed that the transcription level of *Sc*LPMO10G was significantly upregulated, showing a 48.7-fold increase. Therefore, we first expressed *Sc*LPMO10G in *E. coli* to investigate the role in vitro. *Sc*LPMO10G exerts oxidative activity against chitin and promotes in vitro hydrolysis of chitin by chitinase. We also explored the in vivo biological functions of *Sc*LPMO10G for the first time. The LPMO-overexpressing mutant *Sc*ΔLPMO10G(+) strain showed a stronger ability to depolymerise chitin than did the wild-type *Sc*A3(2) strain. These results expand our existing understanding of the highly efficient enzymatic hydrolysis system for chitin utilisation and provide a basis for further research on the biological functions of LPMO.

## Figures and Tables

**Table 1:** The primers used in this study.

Primer	Nucleotide Sequence (5′→3′)	Description
*Sclpmo*10A-qF	GTACTACGTCGGAGGTCAGC	Used to quantify *Sclpmo*10A transcripts
*Sclpmo*10A-qR	GTTGACGTCGATGCAGGC	
*Sclpmo*10B-qF	ACGCTACAACTCCCTCGAC	Used to quantify *Sclpmo*10B transcripts
*Sclpmo*10B-qR	ATCTCGTAGTTCTGGCTGGG	
*Sclpmo*10C-qF	GGACAGCCAGGAGAACTTCT	Used to quantify *Sclpmo*10C transcripts
*Sclpmo*10C-qR	CTCCAGGAGTTCTCCACCG	
*Sclpmo*10D-qF	AAGGGCACGTTCAAGGTCTA	Used to quantify *Sclpmo*10D transcripts
*Sclpmo*10D-qR	AAGGTGACGTCCGAGCAG	
*Sclpmo*10E-qF	ATCTGTTCCGCGGGACAC	Used to quantify *Sclpmo*10E transcripts
*Sclpmo*10E-qR	AGGTTGTGGTTCTGGTTCCA	
*Sclpmo*10F-qF	TGGACTTCGGCGGGTTCAGC	Used to quantify *Sclpmo*10F transcripts
*Sclpmo*10F-qR	GCAGGCGTAGAAGGCGTTGG	
*Sclpmo*10G-qF	CGGCAACGCCTTCTACTCCTG	Used to quantify *Sclpmo*10G transcripts
*Sclpmo*10G-qR	CTTCCCAGACGCCCCATTCG	
*SchrdB*-qF	TCGACTACACCAAGGGCTACAA	Used to quantify *SchrdB* transcripts
*SchrdB*-qR	ACCATGTGCACCGGGATAC	
*Sclpmo*10G-EF	CCGCGCGGCAGCCATATG**ATTGAAGGTCGT**CACGGGTGGGTGACCTCGCCC	Used to amplify *Sclpmo*10G and insert the plasmid pET28a and pGM1190, respectively. The *Nde*I/*EcoR*I site is attached (underlined). Factor Xa cleavage site is indicated in bold.
*Sclpmo*10G-SF	CCGCGCGGCAGCCATATGATGCGCACCAAGAGACGGGTGGC	
*Sclpmo*10G-R	TCGACGGAGCTCGAATTCTCACGCGGCCGCGCAGG	

## Data Availability

Not applicable.

## Supplementary Materials

The following supporting information can be downloaded at: https://www.mdpi.com/article/10.3390/ijms24010275/s1.
