# Supplementary material for: Chitin Biodegradation by Lytic Polysaccharide Monooxygenases from Streptomyces coelicolor In Vitro and In Vivo
Source: Int J Mol Sci. 2022 Dec 23;24(1):275. doi: 10.3390/ijms24010275 (PMC9820598; doi:10.3390/ijms24010275)
Supplement: Supplementary file 1 [file ijms-24-00275-s001.zip › ijms-2110418-supplementary.pdf]

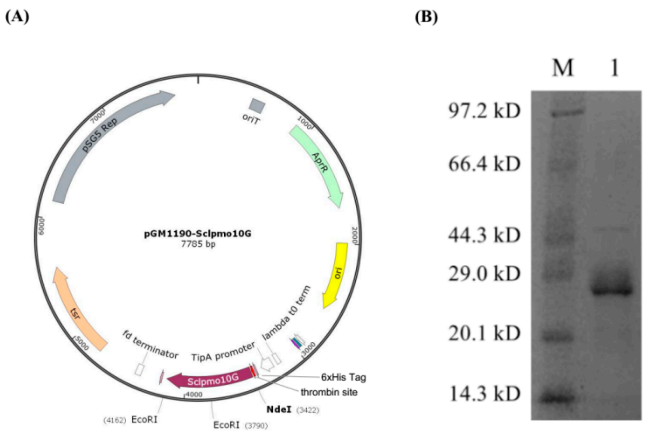

Figure S1. Construction and expression of recombinant plasmid pGM1190- Sc*lpmo*10G. (A) The map of recombinant plasmid pGM1190-Sc*lpmo*10G. (B) SDS-PAGE analysis of recombinant purified *Sc*LPMO10G. Lane M: Protein marker. Lane 1 shows the purified protein *Sc*LPMO10G obtained using a Ni-NTA column.

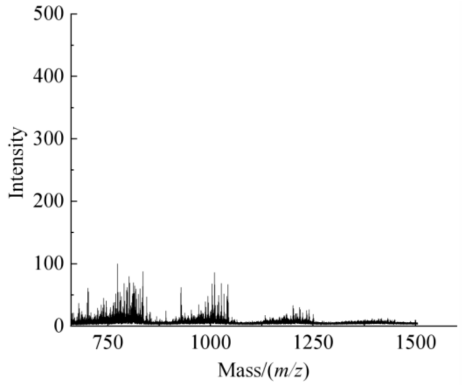

Figure S2. MALDI-TOF MS analysis of PASC incubated with *Sc*LPMO10G. MALDI-TOF spectrum of reaction products ranged from m/z 500 to 1500. The reaction was performed with 10.0 mg/ml PASC, 1.0 mM ascorbic acid and 2 μM *Sc*LPMO10G in 50 mM Tris-HCl buffer (pH8.0) at 30 ℃ for 24 hours.

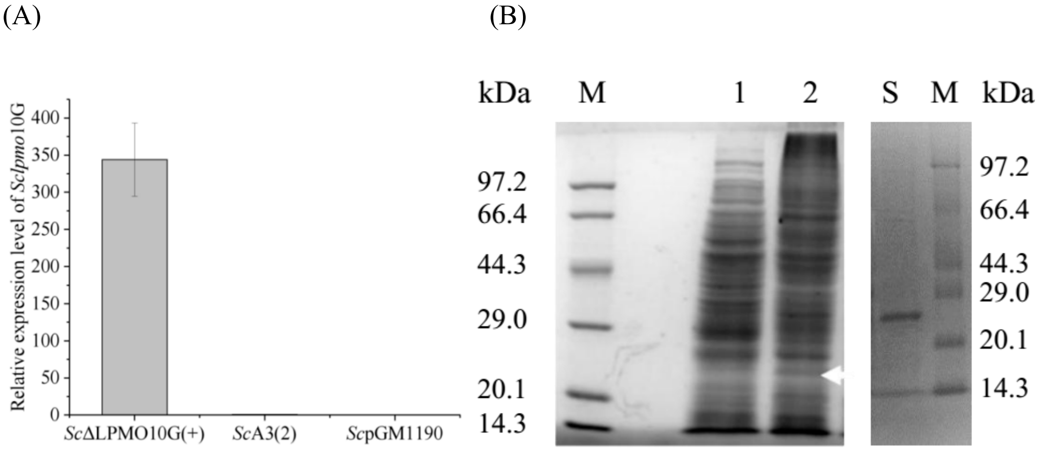

Figure S3. Overexpression of *Sclpmo*10G gene in mutant strain and wild-type strain. (A) The relative transcript level of *Sclpmo*10G in mutant strain *Sc*ΔLPMO10G(+), *Sc*pGM1190 with an empty plasmid pGM1190 and wild-type strain *Sc*A3(2) was analyze by qPCR. (B) SDS-PAGE analysis of extracellular protein in LPMO-overexpressing mutant strain *Sc*ΔLPMO10G(+) and wild-type strain *Sc*A3(2). Lane M: Protein marker. Lane 1 and 2 are total proteins extracted from *Sc*A3(2) and *Sc*ΔLPMO10G(+), respectively. Samples were loaded with the equal amount of protein. Lane S is the purified *Sc*LPMO10G protein. The protein band indicated by white arrow at the corresponding position of *Sc*LPMO10G (25.2 KD) with the same protein mobility as the purified *Sc*LPMO10G protein.

Table S1. Sequence of the combinant plasmid pGM1190- Sc*lpmo*10G

	DNA sequence (5'→3')
pGM1190-<br>Sc*lpmo*10G	ggccgtcgacgacgacgcgtccgcctgcctgcttttggccggcgggccgggccgcggttgtccgccgggtcgg<br>accggtcccttggtcgaccgatgcctcggccacgacgatgctgggcccgaagcggtacgcgaacccgtgccgg<br>gctgcggcggatcgcggcagcaggagctccccgcccgccacggctggctggatggtcgcctcacgcgcccgc<br>aggggtgacggcgtgggtgcggcggtcagccccgtgatcgggcgtcagacggccgctgagggaccgcgacca<br>cccctcccttcccgcctgccgtctttcaccgcccggccggtccgcagacgccgtacggccaggccacggaggatt<br>acgggcagatggcgcccacccggcacccgggctggggcaccctggacagcacgaaggcccgccccactcac<br>cggggcgggccttcgcgttgagctagagttcccgccagcctcgcagagcaggattcccgttgagcaccgccagg<br>tgcgaataagggacagtgaagaaggaacacccgctcgcgggtgggcctacttcacctatcctgcccggctgacg<br>ccgttggatacaccaaggaaagtctacacgaaccctttggcaaaatcctgtatatcgtgcgaaaaaggatggatata<br>ccgaaaaaatcgctataatgaccccgaagcagggttatgcagcggaaaatgcagctcacggtaactgatgccgtat<br>ttgcagtaccagcgtacggcccacagaatgatgtcacgctgaaaatgccggcctttgaatgggttcatgtgcagctc<br>catcagcaaaaggggatgataagtttatcaccaccgactatttgcaacagtgccgttgatcgtgctatgatcgactga<br>tgtcatcagcggtggagtgcaatgtcgtgcaatacgaatggcgaaaagccgagctcatcggtcagcttctcaacctt<br>ggggttaccccggcggtgtgctgctggtccacagctccttccgtagcgtccggcccctcgaagatgggccacttg<br>gactgatcgaggccctgcgtgctgcgctgggtccgggagggacgctcgtcatgccctcgtggtcaggtctggacg<br>acgagccgttcgatcctgccacgtcgcccgttacaccggaccttggagttgtctctgacacattctggcgcctgcca<br>aatgtaaagcgcagcgcccatccatttgcctttgcggcagcggggccacaggcagagcagatcatctctgatccat<br>tgcccctgccacctcactcgcctgcaagcccggtcgcccgtgtccatgaactcgatgggcaggtacttctcctcgg<br>cgtgggacacgatgccaacacgacgctgcatcttgccgagttgatggcaaaggttccctatggggtgccgagaca<br>ctgcaccattcttcaggatggcaagttggtacgcgtcgattatctcgagaatgaccactgctgtgagcgctttgccttg<br>gcggacaggtggctcaaggagaagagccttcagaaggaaggtccagtcggtcatgcctttgctcggttgatccgc<br>tcccgcgacattgtggcgacagccctgggtcaactgggccgagatccgttgatcttcctgcatccgccagaggcg<br>ggatgcgaagaatgcgatgccgctcgccagtcgattggctgagctcataagttcctattccgaagttcctattcgaaa<br>tgaccgaccaagcgacgcccaacctgccatcacgagatttcgattccaccgccgccttctatgaaaggttgggcttc<br>ggaatcgttttccgggacgccggctggatgatcctccagcgcggggatctcatgctggagttcttcgcccacccca<br>aaaggatctaggtgaagatcctttttgataatctcatgaccaaaatcccttaacgtgagttttcgttccactgagcgtca<br>gaccccgtagaaaagatcaaaggatcttcttgagatcctttttttctgcgcgtaatctgctgcttgcaaacaaaaaaac<br>caccgctaccagcggtggtttgtttgccggatcaagagctaccaactctttttccgaaggtaactggcttcagcagag<br>cgcagataccaaatactgtccttctagtgtagccgtagttaggccaccacttcaagaactctgtagcaccgcctacat<br>acctcgctctgctaatcctgttaccagtggctgctgccagtggcgataagtcgtgtcttaccgggttggactcaagac<br>gatagttaccggataaggcgcagcggtcgggctgaacggggggttcgtgcacacagcccagcttggagcgaac<br>gacctacaccgaactgagatacctacagcgtgagcattgagaaagcgccacgcttcccgaagggagaaaggcg<br>gacaggtatccggtaagcggcagggtcggaacaggagagcgcacgagggagcttccagggggaaacgcctg<br>gtatctttatagtcctgtcgggtttcgccacctctgacttgagcgtcgatttttgtgatgctcgtcaggggggcggagc<br>ctatggaaaaacgccagcaacgcggcctttttacggttcctggccttttgctggccttttgctcacatgttctttcctgcg<br>ttatcccctgattctgtggataaccgtattaccgcctttgagtgagctgataccgctcgccgcagccgaacgaccgag<br>cgcagcgagtcagtgagcgaggaagcggaagagcgcccaatacgcaaaccgcctctccccgcgcgttggccg<br>attcattaatgcagctggcacgacaggtttcccgactggaaagcgggcagtgagcgcaacgcaattaatgtgagtt<br>agctcactcattaggcaccccaggctttacactttatgcttccggctcgtatgttgtgtggaattgtgagcggataaca<br>atttcacacaggaaacagctatgaccatgattacgaattgtacgtaggcgtcagctacgatgttccggggactgctg<br>atccggtcagcaggtggaagagggactggattccaaagttctcaatgctgcttgctgttcttgaatggggggtcgttg<br>acgacgacatggctcgattggcgcgacaagttgctgcgattctcaccaataaaaaacgcccggcggcaaccgag

	cgttctgaacaaatccagatggagttctgaggtcattactggaccggatcggggatctgggctgagggagccgac<br>ggcacgcggcggctcacggcgtggcacgcggaacgtccgggcttgcacctcacgtcacgtgaggaggcagcg<br>tggacggcgtcagagaagggagcggaccatgggcagcagccatcatcatcatcatcacagcagcggcctggtg<br>ccgcgcggcagccatatg**atgcgcaccaagagacgggtggccttcgcggtcggggcgctggtcgctccgttg**<br>**ctcgccgtcagcctcccggcgagcacggcgagcgcgcacgggtgggtgacctcgcccggcagccggcagg**<br>**accagtgcgccgcggggatcgtcgactgcggtcagatcaagtacgagccccagagcgtcgagggcccgaa**<br>**gggcctgagcagttgcagcggcggaaacagccagttcgccgaactcgacgacgacggcaagggctggcg**<br>**cgtcacgccggtcggcaccacgcacaccttcacctggcaccacacggcacgccacgccacggccaactgg**<br>**cagtacttcatcggcagccagaagatcgccgaattcgacggccaccgagcgcagcccgccccggacgtga**<br>**gccaccaggtgaacttcggcggcttcaccggccgccagaaggtactggccgtgtggaacgtggcggacac**<br>**cggcaacgccttctactcctgcatcgacgtcaacatcggcggcagcggtgggggcgacgggggcggcgac**<br>**ggcggcggcgacggcgacccgggagcctgcgacgcgcccgggtggagcgccggcagtgtctacagcggc**<br>**ggtgacaccgtctccttcggcggccacacctggcgcgccaagtggtgggtgaccggcgaggagcccggca**<br>**ccaccggcgaatggggcgtctgggaagacctcggagcctgcgcggccgcgtga**gaattcgagctccgtcga<br>caagcttgcggccgcactcgagcaccaccaccaccaccactgagatccggctgctaacccagatctaaagttttgt<br>cgtctttccagacgttagtaaatgaattttctgtatgaggttttgctaaacaactttcaacagtttcagcggagtgagaat<br>agaaaggaacaactaaaggaattgcgaataatttttcacgttgaaaatctccaaaaaaaaaggctccaaaagg<br>agcctttaattgtatcggtttatcagcttgctttcgaggtgaatttcttaaacagcttgataccgatagttgcgccgacaa<br>tgacaacaaccatcgcccacgcataaccgatatattcggtcgctgaggcttgcagggagtcaaaggccgcttttgc<br>gggatctcgtcgaaggcactagaggcctatatgcggggatcgaccgcgcgggtcccggacggggaagagcgg<br>ggagctttgccagagagcgacgacttccccttgcgttggtgattgccggtcagggcagccatccgccatcgtcgcg<br>tagggtgtcacaccccaggaatcgcgtcactgaacacagcagccggtaggacgaccatgactgagttggacacc<br>atcgcaaatccgtccgatcccgcggtgcagcggatcatcgatgtcaccaagccgtcgcgatccaacataaagaca<br>acgttgatcgaggacgtcgagcccctcatgcacagcatcgcggccggggtggagttcatcgaggtctacggcag<br>cgacagcagtccttttccatctgagttgctggatctgtgcgggcggcagaacataccggtccgcctcatcgactcct<br>cgatcgtcaaccagttgttcaaggggggagcggaaggccaagacattcggcatcgcccgcgtccctcgcccggcc<br>aggttcggcgatatcgcgagccggcgtggggacgtcgtcgttctcgacggggtgaagatcgtcgggaacatcgg<br>cgcgatagtacgcacgtcgctcgcgctcggagcgtcggggatcatcctggtcgacagtgacatcaccagcatcgc<br>ggaccggcgtctccaaagggccagccgaggttacgtcttctcccttcccgtcgttctctccggtcgcgaggaggcc<br>atcgccttcattcgggacagcggtatgcagctgatgacgctcaaggcggatggcgacatttccgtgaaggaactcg<br>gggacaatccggatcggctggccttgctgttcggcagcgaaaagggtgggccttccgacctgttcgaggaggcgt<br>cttccgcctcggtttccatccccatgatgagccagaccgagtctctcaacgtttccgtttccctcggaatcgcgctgca<br>cgagaggatcgacaggaatctcgcggccaaccgataagcgcctctgttcctcggacgctcggttgctcgacctcg<br>attcgtcagtgatgatcaacaggacccagaaatggcacgagcccgggattggcctcccgggctcgccgcccccg<br>aaccggcgcagcatgccgagtgaggacgtttgctctgtgacgagcgtacgagagaacagcgcgtcgacaccacc<br>gcgccccgccggaccgaagtgccactgcggcaccccgatcgtccacatccccgggaagcgccccaaaatctact<br>gctcggacgcctgccgaaagcgcgcaaagcgtctcattgcggaccagtcacgcagcgtagcaagcacgactaca<br>gcgggtaatccggacgcgggctttagtagtaagggaaaatctccgtccactgcaggtcagagcgctgtaactgga<br>acgggttctaccccaaacgagggtgccgcgacagccgggcgggaggacccgcgcgacgagcggttcgcccg<br>gcgggaccggcaccagacggtgtcgctgaacgaggcgttcaagggctgcgggacccggctcactgccgggac<br>ggccgagctgatgtggaagccgggcgaggcgacgtggggcaacacgtgccgctgcaacaacgtccacacctg<br>cccgtggtgcatgagccgcatcctggccgtacggggctcgaacgtgcagctcgccgccgacggtctcgcggac<br>gccgggtacgggctgcaccttggcacgaacacgttgcgccacttcgagcgcatggcgttcggcaccgtccgcaa<br>gggaatgcggcacggcctggtcgcggtcttgcacgacggctggaagggcgcgtacggctcgtccggccgccgc

	tggcgcacgatgcgcgacgacttcgggatcatcggctacgagcgggccttcgaggacaccttcggctggggctc<br>cggctggcacctgcactggcacacgctctgggtcacccgcgaggtgctcggcccggacgcccaggccgcgttc<br>cgcgacgcactcgccggagcctgggccgccggggtcgaatccgcgggcggctacaccgtcagcgagacgtgc<br>gaccggcccggttgctcctgtgagggcaagggccacggcaccgacgtgcgcccactcaacggcgcggacgcc<br>gctgacggcgatgcgggcaagcaggcccgctacctctacaaggacggcgacaagaccaagggcggcgtcgcc<br>aagatcggcctggagctggccggacagaacttcaaggccggtcgcggcgacgaccgcatgggaccgctcgacc<br>tgggcgacgcggcggccgccgagctgcagcggcttcgtcggcccggcccgttcgtcgagaagtaccgcgagc<br>gcgagttcggcgtcttccaggtccgcaagcactaccggtcgcagaacctgaaccggctgatcaaggaactcggca<br>tccagcaggacgtccgcaccgaggaggagatcaccgacgacaccgagggcctggtcgccatcgcggtcatccc<br>ggcctacatctggtaccgatacatcgcccgcgtcgccggacgtcgcctcgacctcatcaaggtcgccgagacgta<br>cggcctaccgggcgtccggcggctcgtcgagtcctggggcctggtgtggggcaaggacgtcctggacccgccc<br>gccccggaagcgccggccgcgccgggcgaccttgacgctgaccagatgaggttcgaggtcatgtccgaggag<br>gaagctgctttccgcgaggcgcgccggaaggccaacgaggcccgcacggaggagctggccgcttcgctcgac<br>cgagtgcgtcagccgaagaaggaggcgatccgcccgacgatcagccttcggaagcgcctgaagccgaagccg<br>gtcaccgtcgacgtcaagactcccccgcccggcgccgcgtcgcccgtgtgccgccgctgcaagggcaagctcg<br>ccccggtactccagccctggggccggctccccggagactgcctccgcgtcgacacagcggtcgcgtagggccg<br>ctgagggaccgccatgagaccgccaccccggacatccgggtggcggtctcatggcggtcccggcgcgaaagcc<br>gagggtctgcctgccgtgaggtggccggcggacctgggttactgctggaatcccgcgcccagtccgagcgtccg<br>ccggcgcttttcacctggctccgggtgctcgtcgatgagcacca

The Sc*lpmo*10G gene sequences is marked in bold red.
